# MITF Gain‐of‐Function Mutation in Metastatic Prostate Cancer: A Rare Finding With Potential Therapeutic Significance

**DOI:** 10.1002/cnr2.70588

**Published:** 2026-06-01

**Authors:** Paul J. Pecorin, Nyembezi Dhliwayo, Matthew Demetrious, Ludimila Cavalcante, Veronica Rodriguez‐Bravo, Josep Domingo‐Domenech, Thomas Westbrook

**Affiliations:** ^1^ Division of General Internal Medicine, Department of Medicine Rush University Medical Center Chicago Illinois USA; ^2^ Division of Hematology, Medical Oncology and Cell Therapy, Department of Medicine Rush University Medical Center Chicago Illinois USA; ^3^ Department of Pathology Rush University Medical Center Chicago Illinois USA; ^4^ Division of Hematology & Oncology, Department of Medicine University of Virginia Charlottesville Virginia USA; ^5^ Department of Urology Mayo Comprehensive Cancer Center Rochester Minnesota USA

## Abstract

**Background:**

Prostate cancer is the most common non‐cutaneous malignancy in men and remains a leading cause of cancer‐related mortality, particularly in aggressive variants. These tumors are usually associated with alterations in TP53, RB1, and PTEN. The microphthalmia‐associated transcription factor (MITF) has been implicated in several malignancies, but its role in prostate cancer is less defined.

**Case:**

We report the first case of metastatic prostate adenocarcinoma harboring a gain‐of‐function MITF E318K mutation. The patient presented with high tumor burden, rapid progression, and evidence of tumor heterogeneity. The E318K mutation, previously linked to melanoma and renal cell carcinoma, enhances MITF transcriptional activity and may contribute to aggressive disease behavior.

**Conclusion:**

This case expands current understanding of MITF in prostate cancer and raises important questions regarding its prognostic and therapeutic significance.

## 1 Background

Prostate cancer is one of the most commonly diagnosed malignancies among men in the United States, with significant morbidity and mortality, particularly in aggressive variants that present with concomitant metastasis at diagnosis. Recent studies have implicated the microphthalmia‐associated transcription factor (MITF) in the pathogenesis of prostate cancer. In this report, we present the first documented case of gain‐of‐function MITF mutation that may enhance aggressive prostate cancer behavior.

## 2 Introduction

Prostate cancer is the most common non‐cutaneous malignancy among men in the United States, with a lifetime risk of 1 in 8, and it accounts for 11% of all cancer‐related deaths in men ‐underscoring its clinical impact [1]. Localized prostate adenocarcinoma is successfully managed with surgery, radiation, or active surveillance. However, a subset of patients experience progression to metastatic disease, where multiple therapeutic strategies including anti‐androgen and taxane therapy, among others, improve survival [2]. In this context, aggressive prostate cancer variants—characterized by undifferentiated phenotype and poor response to chemo‐hormonal therapies—represent a smaller yet highly challenging subset of cases [3]. These aggressive variants are molecularly characterized by co‐occurring genetic alterations in canonical drivers such as *TP53*, *RB1*, and *PTEN*. However, alternative genetic alterations may also contribute to the aggressive behavior of these prostate cancer variants.

The microphthalmia‐associated transcription factor (MITF) has been implicated in the pathogenesis of multiple malignancies, including renal cell carcinoma [4, 5] and melanoma [6, 7, 8, 9]. Interestingly, in the context of melanoma, both MITF‐gain [10] and ‐loss [7, 8, 9] of function have been shown to contribute to the pathobiology of melanoma. More recently, MITF has been associated with prostate cancer pathogenesis. Low MITF expression has been linked to tumor initiation [10] as well as to castration resistance and immune evasion in advanced stages of the disease [11].

This report describes the first known case of metastatic prostate adenocarcinoma harboring a pathogenic gain‐of‐function MITF E318K mutation identified at diagnosis. While aggressive prostate cancer is typically driven by alterations in TP53, RB1, and PTEN, gain‐of‐function mutations in MITF have not previously been reported in this disease. The patient presented with de novo metastatic disease, rapid progression, and marked tumor heterogeneity prior to substantial treatment exposure. Given the established oncogenic role of MITF E318K in melanoma and renal cell carcinoma, this finding raises the possibility that MITF may act as a driver of tumor behavior in aggressive prostate cancer.

## 3 Case

A 57‐year‐old man presented to the Rush University Medical Center emergency department (ED) in February 2024 with worsening pelvic and hip pain (Table 1). Imaging revealed pelvic and retroperitoneal lymphadenopathy, with the largest node measuring 1.2 × 1.2 cm in the para‐aortic region. He was empirically treated with ciprofloxacin for presumed prostatitis, leading to symptomatic improvement. However, subsequent testing by his primary care provider revealed a markedly elevated prostate‐specific antigen (PSA) level of 107.0 ng/mL (reference range 0.0–0.4 ng/mL), prompting referral to urology.

**TABLE 1:** Case timeline.

2/13/2024	Presented with pelvic pain. Imaging showing retroperitoneal lymphadenopathy. Treated for presumed prostatitis
3/2024	Workup sent with PSA 107 ng/mL. MRI Prostate shows sPI‐RADS 5 lesion
3/26/2024	Biopsy shows Gleason 8–9 adenocarcinoma
4/5/2024	Hospitalized with acute renal failure, requiring hemodialysis, transurethral resection of the prostate and nephrostomy tubes
4/8/2024	Started on bicalutamide and degarelix
4/17/2024	Bronchoscopy confirmed pulmonary metastases from prostate primary
5/16/2024	Transitioned to darolutamide and leuprorelin
5/24/2024	DOTATATE showed diffuse avid disease
5/31/2024	Treatment escalated, docetaxel and denosumab added
7/25/2024	Carboplatin added to chemotherapy regime due to PSA plateu
11/7/2024	Completed carboplatin and docetaxel
6/17/2025	PSA nadir to 3.08 ng/mL
3/27/2026	CT showing interval progression in rib lesions and multiple enlarged retroperitoneal and pelvic lymph nodes
4/7/2026	Resumed on carboplatin and docetaxel

An MRI of the prostate revealed a PI‐RADS 5 lesion spanning from the base to the apex of the prostate, with invasion of the posterior bladder and mesorectal fat without rectal wall involvement. Findings also included lymphadenopathy suggestive of nodal metastasis and hyperintensity in the bilateral ischial tuberosities, pubic symphysis, and acetabular margins, raising concern for bone metastases (Figure 1). Bone metastases were later confirmed with a nuclear bone scan (Figure 2). Prostate biopsy confirmed adenocarcinoma in all 12 cores, with Gleason scores of 8–9, establishing a diagnosis of metastatic prostate adenocarcinoma (Figure 3A,B).

**FIGURE 1:**
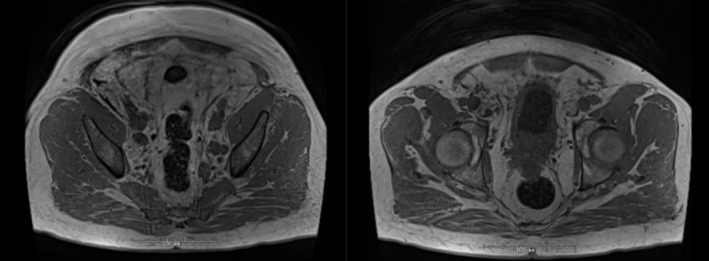
initial diagnostic prostatic MRI showing a large 5.5 × 4.3 × 3.9 cm PI‐RADS‐5 lesion extending from the base to apex of the prostate with invasion of the posterior urinary bladder wall and anterior mesorectal fat, abutting the anterior rectal wall without definite invasion. Multiple enlarged pelvic lymph nodes also noted.

**FIGURE 2:**
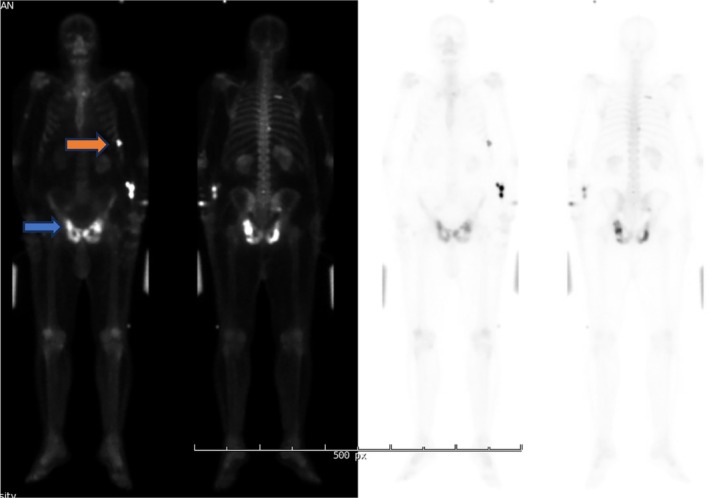
Initial nuclear bone scan showing heterogenous radiotracer uptake involving the bilateral paramidline pelvic bones (blue arrow), focal uptake in the posterior arch of the left fourth rib (orange arrow), and indeterminate uptake in the left ninth costovertebral joint.

**FIGURE 3:**
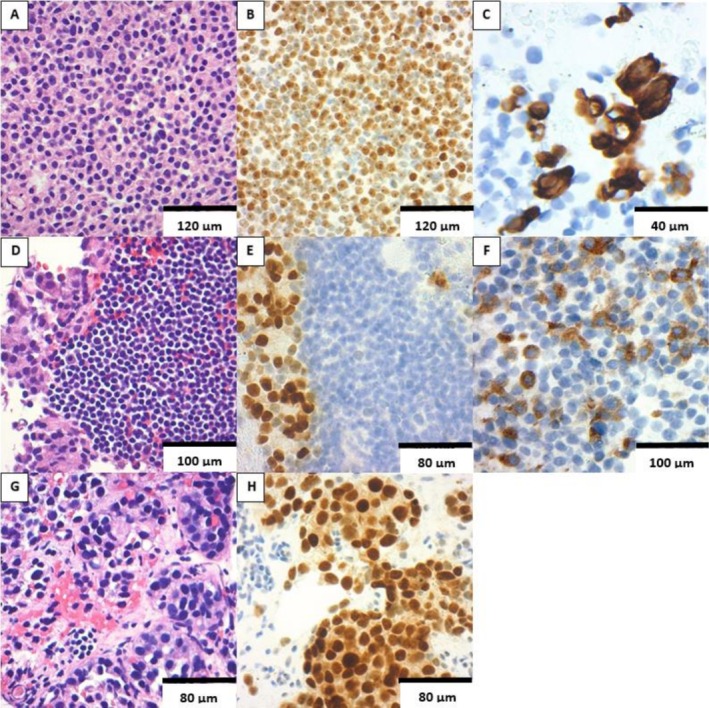
This figure presents the histopathological characteristics of prostatic adenocarcinoma with a MITF mutation. Prostate chips with H&E staining reveal irregularly shaped glands that are fused, accompanied by a loss of normal glandular differentiation (A); NKX3.1 immunohistochemical staining shows retained expression (B). Metastatic tumor cells with rare but strong expression of CK7 in a lymph node biopsy (C). Station 7 lymph nodes with H&E staining and NKX3.1 expression demonstrate infiltrative metastatic involvement, characterized by enlarged, pleomorphic nuclei with prominent nucleoli (D and E). Variable expression of synaptophysin among metastatic cells in a lymph node (E). Retroperitoneal lymph nodes reveal extensive metastatic deposits with tumor cells exhibiting abundant eosinophilic cytoplasm (G); NKX3.1 staining underscores the aggressive nature of the tumor by highlighting nuclear enlargement of the tumor cells compared to the adjacent lymphocytes (H).

Before attending outpatient appointments, the patient returned to the ED with progressive dyspnea, fatigue, and intermittent hematuria. Laboratory evaluation revealed acute renal failure, Cr 29 mg/dL (reference range 0.75–1.20 mg/dL), BUN 148 mg/dL (reference range 22–29 mg/dL), K 8 mmol/L (reference range 3.4–5.3 mmol/L), bicarbonate < 5 mmol/L (reference range 22–29 mmol/L), anion gap 27 mmol/L (reference range 8–16 mmol/L), phosphorus 15.4 mg/dL (reference range 2.5–4.6 mg/dL), severe metabolic acidosis, and anemia, hemoglobin 8 g/dL (reference range 13.5–17.5 g/dL). CT pulmonary angiogram showed enlarged mediastinal and hilar lymph nodes along with multiple pulmonary nodules. A Foley catheter was placed for significant hematuria with clot retention, and hemodialysis was initiated.

His course was complicated by recurrent clot obstruction of the Foley catheter, necessitating cystoscopy with transurethral resection of the prostate, clot evacuation, and fulguration of prostatic bleeding, followed by bilateral percutaneous nephrostomy (PCN) placement. Renal function recovered, allowing hemodialysis to be discontinued after 6 days. He was started on bicalutamide 50 mg daily and degarelix 120 mg once and discharged with follow‐up plans, including oncology consultation and bronchoscopy to evaluate pulmonary findings.

Post‐discharge workup included next‐generation sequencing with the Tempus xT^(c)^, xF^(c)^, and xG^(c)^ platforms. These revealed PD‐L1 expression < 1%, a TMPRSS2‐ERG rearrangement, a TP53 loss of function mutation, PTEN copy number loss, a CCND1 gain‐of‐function mutation, and a tumor mutational burden of 4.2. Tempus xG+^(c)^ identified a heterozygous pathogenic E318K MITF mutation (Table 2). His PSA, which had peaked at 203 during hospitalization, decreased to 27 after 5 weeks of therapy with bicalutamide and degarelix. Bicalutamide was initiated during acute hospitalization and utilized transiently to allow for immediate androgen blockade in the setting of critical illness and formulary accessibility. Following clinical stabilization, systemic therapy was transitioned from bicalutamide to darolutamide 600 mg two times per day, and leuprorelin 22.5 mg every 3 months was initiated in replacement for degarelix.

**TABLE 2:** Summary of genomic alterations detected in the patient's tumor, as reported by Tempus xT, xF, and xG platforms.

Test	Platform	Mutation	VAF	Result
PDL1 TPS	Tempus xT			< 1%
PDL1 CPS	Tempus xT			< 1
TMPRSS2‐ERG	Tempus xT	[TMPRSS2‐ERG] Chromosomal rearrangement		
TP53	Tempus xT	p.M246V—c.732_736delinsGGGCG (Missense variant—LOF)	44.90%	
PTEN	Tempus xT	Copy number loss		
CCND1	Tempus xF and xT	p.P287S—c.859C > T (Missense variant—GOF)	0.50%	
TMB	Tempus xT			4.2 mutations/megabase
MITF	Tempus xG	p.(Glu318Lys) (GAA > AAA): c.952 G > A in exon 9 of the MITF gene		

*Note:* Variants are annotated with protein changes, variant allele frequencies (VAF), and type of alteration where applicable.

Further evaluation of mediastinal and hilar lymphadenopathy, as well as pulmonary nodules, included bronchoscopy with biopsy of station 7 and 11 lymph nodes. Histopathology confirmed metastatic prostate adenocarcinoma with strong diffuse expression of NKX3.1 and PSA (Figure 3C,D). Additionally, there was faint positive staining for CK 7 and synaptophysin; however, there was no evidence of neuroendocrine features on hematoxylin and eosin (H&E) staining. There was concern for a secondary carcinoid malignancy, and a PET‐DOTATATE scan was performed which revealed diffuse DOTATATE avid metastatic lesions but no primary carcinoid tumor. DOTATATE avidity was most notable in the axial and appendicular skeleton; however, there were also avid abdominopelvic and thoracic lymph nodes (Figure 4). Although biopsied lymph nodes did not demonstrate neuroendocrine differentiation, the DOTATATE scan revealed lesions likely to be neuroendocrine tumors based on their high avidity. This suggests the presence of significant tumor heterogeneity, a common feature of aggressive disease. Retroperitoneal lymph nodes were later biopsied and confirmed to be metastatic prostate adenocarcinoma without neuroendocrine features (Figure 3E,F).

**FIGURE 4:**
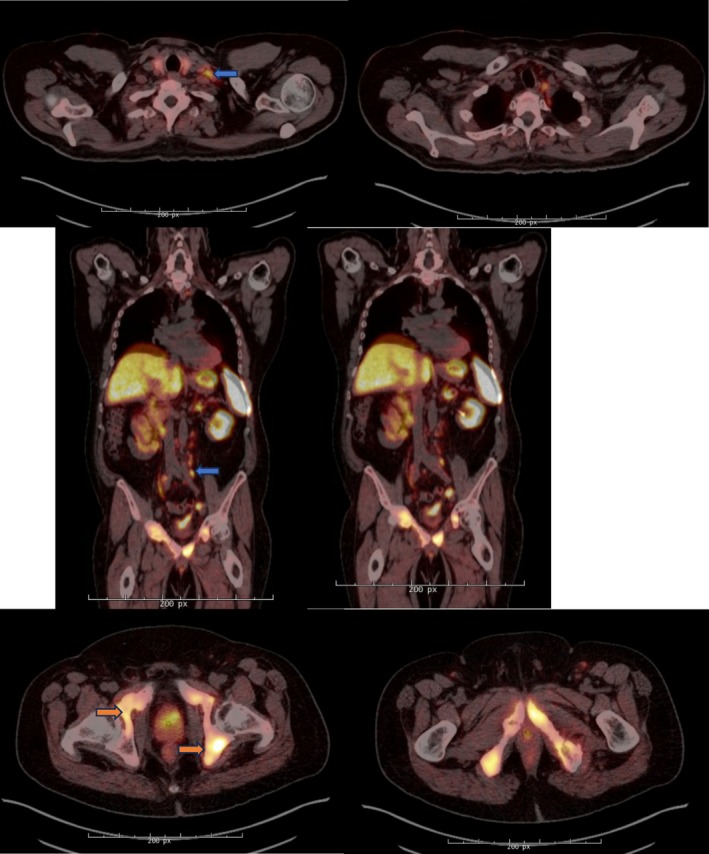
Initial diagnostic PET‐DOTATE showed innumerable DOTATATE avid lymph nodes throughout the neck, chest, abdomen and pelvis (representative lesions shown with blue arrows), as well as innumerable foci of DOTATATE avid osseous metastatic lesions, including the left frontal calvarium, manubrium and pelvic bones (representative areas shown with orange arrows) and pathologic fracture of the right superior inferior pubic rami.

Following confirmation of prostate cancer as etiology of metastatic lesions, denosumab 120 mg every 3 months and docetaxel 75 mg/m^2^ every 3 weeks were added to the current combination of androgen‐receptor signal inhibition and androgen deprivation therapy with darolutamide and leuprorelin respectively. This stepwise escalation reflects a transition to guideline‐directed treatment intensification following clinical stabilization, after initial therapy was initiated during acute illness.

While receiving cycle 1 of docetaxel, he experienced an infusion reaction with facial flushing and lightheadedness. This resolved with diphenhydramine, famotidine, and decreasing infusion rate. Despite pre‐medication, he had a repeat infusion reaction during cycle 2, resolved with further rate decrease. He did not experience additional infusion reactions going forward.

Due to a PSA plateau at 34.97 ng/mL after 3 cycles of chemotherapy, carboplatin was added (AUC 5 mg/mL per minute). After completion of 8 cycles of chemotherapy, staging scans showed interval improvement in lymphadenopathy but new scattered sclerotic bony lesions. PSA has downtrended and normalized to 3.08 ng/mL. He remained on denosumab, leuprorelin, and darolutamide with continued surveillance showing stable imaging and PSA.

Unfortunately, CT scan 17 months later demonstrated progression of rib lesions and significant retroperitoneal and pelvic lymphadenopathy. This was associated with a PSA increase to 44.02 ng/mL prompting a resumption of docetaxel and carboplatin due to prior durable response.

## 4 Discussion

Aggressive variant prostate tumors are characterized by genetic alterations in canonical oncogenes and tumor suppressors. The identification of a pathogenic MITF mutation in this patient with metastatic prostate adenocarcinoma represents a rare and potentially clinically significant finding in the molecular landscape of prostate cancer.

Although both gain and loss of MITF function have been found to contribute to cancer development, to date only MITF loss‐of‐function has been implicated in prostate cancer pathogenesis. Here we report for the first time a gain‐of‐function MITF mutation potentially contributing to a tumor's aggressive phenotype. Of note, the MITF E318K missense mutation detected in this patient is associated with a higher incidence of melanoma and renal cell carcinoma [12, 13]. This mutation severely impairs MITF SUMOylation, enhancing MITF protein stability and transcriptional activity, leading to increased clonogenicity and invasiveness of cancer cells (Figure 5).

**FIGURE 5:**
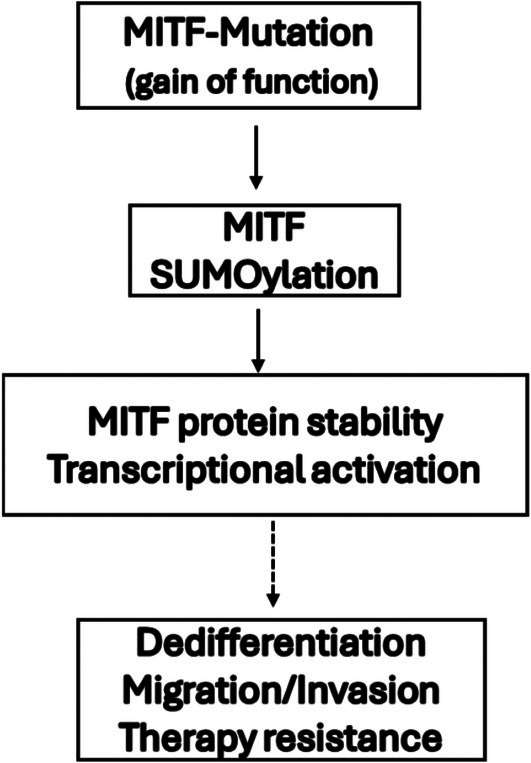
Proposed mechanism of MITF gain of function leading to aggressive behavior in prostate carcinoma. MITF E318K mutation leads to increased MITF protein expression and transcriptional activity inducing dedifferentiation, invasion, and therapy resistance.

This case‐report underscores the potential clinical impact of MITF mutations in cancer. The patient's aggressive disease course, characterized by high tumor burden, extensive lymphadenopathy, evidence of tumor heterogeneity with suspected neuroendocrine positive lesions, and rapid progression, aligns with the proposed consequences of MITF gain‐of‐function in combination with known pathologic alterations such as TP53 mutation and PTEN loss. Additionally, the presence of tumor heterogeneity, including neuroendocrine features, raises the possibility that this tumor may exhibit features of lineage plasticity rather than a defined phenotype.

Immunohistochemical evaluation demonstrated weak CK7 expression and faint synaptophysin without definitive neuroendocrine differentiation. While CK7 is not a typical marker for prostate adenocarcinoma, emerging evidence suggests that KRT7 (CK7) expression may define a subset of aggressive prostate cancers. Recent single cell RNA sequencing analyses have identified a KRT7 positive prostate cancer characterized by low androgen receptor signaling and absence of neuroendocrine features [14].

In this context, the weak CK7 positivity observed in conjunction with discordant neuroendocrine features of DOTATATE avidity without confirmatory histology may reflect tumor lineage plasticity. This may serve as a transitional state between adenocarcinoma and an alternative neuroendocrine phenotype that has been recognized as a mechanism of aggressive behavior and tumor heterogeneity in advanced prostate cancer.

The additional genomic alterations in this patient's tumor, including TP53 mutation and PTEN loss, are well known contributors to neuroendocrine differentiation and aggressive behavior [15, 16]. However, RB1 loss, which has been implicated in both treatment‐emergent and de novo neuroendocrine prostate cancer, is notably absent in this case [17]. In addition, the patient's aggressive clinical features predated exposure to anti‐androgen therapy, which is uncommon.

In this context, the presence of an MITF mutation raises the possibility that it may act as a cooperative modifier of tumor behavior. While we do not suggest that MITF independently drives the observed phenotype, its interaction with TP53 and PTEN loss could plausibly contribute to phenotypic plasticity. This remains speculative in the absence of functional validation.

Beyond its prognostic significance, MITF alterations present a compelling avenue for therapeutic stratification. MITF downregulation has been linked to increased eukaryotic translation initiation factor 3B (eIF3B) expression, which regulates cell growth and protein synthesis. Specifically, this leads to increased androgen receptor (AR) translation and decreased major histocompatibility complex 1 (MHC‐1) translation conferring castration resistance and immune evasion in prostate cancer [11]. While this highlights the tumor‐suppressive role of MITF in prostate cancer, our case suggests that MITF gain‐of‐function mutations such as E318K may drive aggressive variants with heterogeneous differentiation, which respond poorly to existing anti‐androgen and chemotherapy treatment options.

Despite the potential biologic relevance of MITF alterations, there are currently no approved therapies that directly target MITF. As a transcription factor, MITF has historically been difficult to target pharmacologically. However, preclinical studies, primarily in melanoma and renal cell carcinoma, have explored strategies to modulate MITF activity [18, 19, 20]. While these agents remain investigational, they highlight a potential avenue for future therapeutic development. In this context, the identification of a gain‐of‐function MITF mutation in prostate cancer may have implications for future treatment strategies, although clinical applicability remains undefined. Therefore, additional research exploring the restoration of MITF function or inhibition of its downstream signaling pathways could be highly beneficial for cancer patients with similar molecular profiles.

## 5 Conclusion

This case of a 57‐year‐old man with metastatic prostate adenocarcinoma and a rare gain‐of‐function E318K MITF mutation highlights the evolving complexity of prostate cancer genomics. The presence of an MITF mutation in the context of aggressive disease suggests that MITF may play a crucial role in tumor progression and treatment resistance. Importantly, this case suggests that MITF may function as a cooperative modifier of tumor behavior in the setting of established drivers such as TP53 and PTEN loss, rather than acting as a sole oncogenic driver. Additionally, this case is further unique in the identification of gain‐of‐function MITF mutation at diagnosis, prior to therapeutic pressure. Given its emerging significance, further studies are warranted to explore the clinical implications of MITF mutations in prostate cancer.

As next‐generation sequencing becomes more widely utilized, identifying rare mutations such as those affecting MITF will refine our understanding of prostate cancer biology and guide the development of targeted therapies. A key takeaway from this case is that uncommon genomic alterations, even when not classically associated with prostate cancer, may have meaningful biological and potential therapeutic relevance. This case underscores the need for continued research into MITF and other novel molecular drivers, which may ultimately lead to more personalized and effective treatment strategies for patients with advanced prostate cancer.

## Author Contributions

**Paul J. Pecorin:** writing – original draft, writing – review and editing, conceptualization, visualization. **Nyembezi Dhliwayo:** writing – original draft, conceptualization. **Matthew Demetrious:** writing – review and editing, visualization. **Ludimila Cavalcante:** writing – review and editing, supervision. **Veronica Rodriguez‐Bravo:** writing – review and editing, supervision. **Josep Domingo‐Domenech:** writing – review and editing, supervision. **Thomas Westbrook:** conceptualization, writing – review and editing, supervision, writing – original draft.

## Funding

The authors have nothing to report.

## Disclosure

The authors have nothing to report.

## Ethics Statement

This case has been done in accordance to the Wiley ethics and integrity guidelines and has been performed in an ethical and responsible way, with no research misconduct, which includes, but is not limited to data fabrication and falsification, plagiarism, image manipulation, unethical research, biased reporting, authorship abuse, redundant or duplicate publication, and undeclared conflicts of interest.

## Consent

Written informed consent was obtained from the patient, including consent to publish.

## Conflicts of Interest

The authors declare no conflicts of interest.

## Data Availability

No new data was generated.
